# Assessment of Dental Students’ and General Dentistry Residents’ Knowledge Regarding the Management of Anaphylactic Shock in the Dental Practice: A Single-Centre Study in Romania

**DOI:** 10.3390/dj14020075

**Published:** 2026-02-02

**Authors:** Alice Murariu, Elena-Raluca Baciu, Cezara Andreea Onică, Dragoș Nicolae Frățilă, Răzvan Constatin Brânzan, Livia Bobu, Cezar Ilie Foia, Costin Iulian Lupu

**Affiliations:** Grigore T. Popa University of Medicine and Pharmacy Iasi, 700115 Iasi, Romania; alice.murariu@umfiasi.ro (A.M.); cezara-andreea.onica@umfiasi.ro (C.A.O.); dragos.fratila@umfiasi.ro (D.N.F.); razvan-constantin.branzan@umfiasi.ro (R.C.B.); livia.bobu@umfiasi.ro (L.B.); foia.cezar-ilie@d.umfiasi.ro (C.I.F.); iulian.lupu@umfiasi.ro (C.I.L.)

**Keywords:** anaphylaxis, dental emergency, dental students, knowledge, residents

## Abstract

**Background/Objectives**: Anaphylaxis is a rare occurrence in dental practice, yet when it happens, it demands swift management, as untreated cases can be fatal. The aim of this study is to evaluate the level of knowledge among dental students and residents regarding the symptoms and management of anaphylactic emergencies in dental surgery. **Methods**: The study involved a sample of 236 students from the 3rd and 5th years, and residents in their 1st and 2nd years of the General Dentistry programme at the Faculty of Dental Medicine in Iași, Romania. The response rate to the invitation was 85.8%. Knowledge was assessed using a self-administered questionnaire consisting of 18 questions organised into three sections, which were tested for internal consistency, yielding a Cronbach’s alpha value of 0.731. **Results**: Statistically significant differences in the responses provided by the three categories of participants were observed for the following items: management of patients with an allergic background (*p* = 0.033), factors aggravating allergic predisposition (*p* = 0.001), the correct dose of epinephrine (*p* = 0.001), secondary medication (*p* = 0.001), and the timing of treatment initiation (*p* = 0.009). Questions where answers indicated moderate to low levels of knowledge (25–50% correct answers) concerned the therapeutic approach for patients with an allergic background, the site of adrenaline administration, and secondary medication. **Conclusions**: Overall, it can be observed that students demonstrated a high level of knowledge in questions related to the symptomatology of anaphylaxis and the therapeutic management of allergic patients, whereas residents showed better performance in questions addressing the therapeutic management of anaphylaxis. However, significant knowledge gaps were identified across all participant categories, suggesting that there must be periodic supplementary training.

## 1. Introduction

Allergies pose an increasingly significant health challenge affecting people of all ages. In recent years, the number of allergy cases has steadily increased due to various factors, including environmental pollution and changes in diet and lifestyle. As a result, allergies represent a potential global health risk, impacting the quality of food, water, and air [1]. The prevalence of allergies is widespread among both children and adults, affecting approximately 20–30% of the population. According to specialist literature, food allergies can persist throughout a person’s life, with a reported prevalence of 19.9% in Europe [2]. Globally, prevalence varies across regions; however, when viewed as a global health concern, allergies are estimated to affect about 8% of children and 10% of adults. In developed countries and urban areas, there is a noticeable rising trend in allergy prevalence, likely due to the effects of environmental pollution [1,3]. Symptoms of allergies can range from mild skin reactions and seasonal rhinitis to severe cases of anaphylaxis, which can be life-threatening.

The World Allergy Organization defines an allergy as a hypersensitivity reaction caused by specific immunological mechanisms in response to a usually harmless environmental substance called an allergen [4].

Allergic reactions can be classified as type I (immediate hypersensitivity), which occur quickly within minutes or hours after contact with the causal antigen and are mainly mediated by immunoglobulin E antibodies. These antibodies bind to mast cells, inducing their degranulation and the subsequent release of histamine, leukotrienes, and other mediators [4]. The reactions may be localised, resulting in allergic contact urticaria, rhinoconjunctivitis, and asthma, or generalised and potentially life-threatening, leading to anaphylaxis.

Type IV reactions, also known as delayed hypersensitivity, generally take 24 to 48 h after antigen exposure to manifest. This cell-mediated response relies on a complex interaction between antigen-specific T cells and macrophages with dermal and epidermal cells, illustrating the pathogenic mechanism behind allergic contact dermatitis [5,6].

Dental practice involves the use of various materials and products that may trigger adverse reactions and is associated with numerous procedures carrying a high risk of hypersensitivity reactions [7].

Numerous studies in the literature address agents responsible for hypersensitivity reactions in dental practice, with the most commonly implicated being: antibiotics such as penicillin and cephalosporins, antiseptics such as chlorhexidine, anti-inflammatory drugs, local anaesthetics, latex items, endodontic materials, impression materials, acrylics used in dental prostheses, and metals such as nickel, chromium, and cobalt [8,9,10,11,12,13,14,15,16,17,18].

Hypersensitivity in its acute, severe, and potentially deadly form is known as anaphylaxis [19]. According to the 2020 World Allergy Organization guideline on anaphylactic shock, anaphylaxis is a severe systemic hypersensitivity reaction that usually develops rapidly and may be potentially fatal [8].

The population-based incidence of anaphylaxis in Europe is estimated at 1.5–7.9 per 100,000 person-years [20]. Studies indicate that about 0.3% of the European population has experienced anaphylaxis at some stage in their lives. These figures differ based on age, geographical location, and exposure patterns [21].

The initial signs of acute episodes include mucosal or cutaneous symptoms such as urticaria, angioedema, and facial erythema, along with indicators of respiratory distress like bronchospasm, wheezing, hypotension, and/or organ dysfunction [22]. The consequences of anaphylaxis can be severe, potentially leading to death from respiratory obstruction, cardiovascular collapse, or both. Additionally, since up to 20% of anaphylactic cases lack cutaneous symptoms, delays in medical treatment are possible, which can worsen patient outcomes [23].

The onset of anaphylaxis following stings or allergen injections is usually rapid: 70% occur within less than 20 min, and 90% within less than 40 min [24].

When anaphylactic shock is suspected, it is essential to start treatment quickly if respiratory or cardiovascular symptoms are evident, even without cutaneous signs, and to contact emergency medical services immediately. Additionally, after managing the acute phase of an anaphylactic episode, it is vital to confirm the diagnosis and determine the specific trigger responsible for the reaction [25].

There are several challenges and barriers in managing anaphylactic shock: many dentists are unaware of the existing protocols for its management. Furthermore, in developing countries, essential medicines and treatment facilities may be unavailable, and access to adrenaline auto-injectors could be limited. When combined with limited experience and training, these factors can result in poor outcomes [26].

Dentists should not only be aware of such reactions but also know how to manage immediate and severe responses like anaphylaxis and avoid misdiagnosing hypersensitivity reactions. In elderly patients, the combination of advanced age, polypharmacy, and an allergic predisposition—along with dental procedures such as lidocaine anaesthesia—may elevate the risk of medical emergencies like anaphylaxis [27].

In a systematic review on this subject, Kazempour [25] found that practitioner preparedness is inadequate, revealing that most dentists do not receive sufficient training in anaphylaxis management protocols, airway stabilisation techniques, or the proper use of adrenaline.

Therefore, this study aims to assess and compare the knowledge of dental students at different stages of training and residents at the Faculty of Dental Medicine in Iași regarding the management of anaphylactic episodes in dental practice. The null hypothesis states that there are no statistically significant differences in knowledge levels related to anaphylaxis management among participants from different academic levels.

## 2. Materials and Methods

### 2.1. Research Design

The cross-sectional study was carried out between June 2025 and November 2025 at the Faculty of Dental Medicine in Iași, Romania, involving a final sample of 236 dental students from 3rd and 5th years and residents in their 1st and 2nd years of training in the General Dentistry speciality. The study received approval from the Ethics Committee of the “Grigore T. Popa” University of Medicine and Pharmacy Iași, approval no. 612/19.06.2025. All participants signed informed consent and were informed about the purpose of the study, being assured of the anonymity of their answers, and that participation was voluntary.

### 2.2. The Study Group

The sampling method used for participant selection was convenience sampling. Of the total 275 students in the 3rd and 5th years and residents invited to complete the questionnaire, 257 responded; subsequently, 21 questionnaires were invalidated due to issues such as incomplete responses or incorrect completion. Ultimately, the study participation rate was 85.8%.

### 2.3. The Study Instrument

To develop the questionnaire, a literature review was carried out focusing on dental professionals’ knowledge regarding the management of anaphylactic shock [28,29]. Questions from studies containing validated questionnaires were considered. In particular, the instrument proposed by Baççıoğlu [28], which had been previously validated and used in earlier research, served as a reference for the present study.

Additionally, the data were compared with information published by the European Academy of Allergy and Clinical Immunology, Food Allergy, Anaphylaxis Guidelines Group [30].

The questionnaire was prepared in Romanian. The original English version was translated into Romanian by two language specialists, followed by a back-translation into English. The final version included 18 questions. Before starting the study, a pilot test was conducted with a small group of participants (20 residents and graduates) to evaluate question clarity and understanding. After minor necessary adjustments, the final questionnaire was finalised, with an estimated completion time of 7 min. The final questionnaire was tested for internal consistency and achieved a Cronbach’s alpha coefficient of 0.731, which is considered acceptable [31].

The questionnaire consisted of three sections. The first section collected general demographic information: age, sex, and year of study. The second section contained four questions (Q1–Q4) addressing behaviour towards patients with allergic histories, early symptoms of anaphylaxis, substances commonly used in dentistry that may trigger anaphylactic shock, and factors that may worsen a patient’s allergic predisposition. The third section included fourteen questions (Q5–Q18) covering the therapeutic management of anaphylaxis—namely, the drug of choice, timing and steps for initiating treatment, correct dosage, route and site of administration, secondary medications, and knowledge regarding the epinephrine auto-injector. In this section, the last two questions (Q17, Q18) referred to practical experience and self-confidence in the ability to apply emergency measures for anaphylaxis.

The questionnaire was distributed to third- and fifth-year dental students during course sessions and to first- and second-year General Dentistry residents during their practical training.

To classify the knowledge level (KL), the following criteria were applied [32]:

- Excellent: 100–90% correct answers;
- Good: 90–70%;
- Moderate: 70–50%;
- Low: 50–25%;
- Very low: below 25%.

### 2.4. Data Analysis

The Statistical Package for Social Sciences (SPSS Inc., Chicago, IL, USA, version 26 for Windows) was utilised for the statistical analysis. Frequency, percentage, and cross-tabulation were employed for descriptive data analysis. Differences between groups were determined using the Chi-square test, with a significance level of 5% (*p* < 0.05). The non-parametric Kruskal–Wallis test was conducted to compare the knowledge levels of the three participant categories.

## 3. Results

### 3.1. Study Participants

A total of 236 participants participated in the study, with the following distribution: 97 (41.1%) were third-year Dental Medicine students, 92 (39%) were fifth-year Dental Medicine students, and 47 (19.9%) were resident doctors specialising in General Dentistry. The mean age was 24.01 ± 0.39 years.

The demographic characteristics of the study participants, including gender and age distribution, are presented in Table 1.

### 3.2. Participants’ Knowledge About the Characteristics of Anaphylaxis-Clinical Aspects

The responses provided by the study participants to questions Q1–Q4 (Section 2) in this section are shown in Table 2.

For Question 1, the correct answer—“I refer the patient for a specialist consultation in the field of allergology”—was recognised by 52.6% of third-year students, 47.8% of fifth-year students, and 48.9% of residents. Table 2 shows that a significant proportion of respondents (44.3% of third-year students, 45.7% of fifth-year students, and 40.4% of residents) believed they could personally perform an allergy test in the dental office, which is incorrect (*p* = 0.033). The correct answers to Question 2 were facial oedema, dyspnoea, and pruritus. These symptoms were recognised by over 60% of all participants, although with some variations: dyspnoea and pruritus were identified more frequently by residents, while facial oedema was more often recognised by third-year students. Lidocaine and penicillin are substances capable of triggering an anaphylactic shock (Q3), with correctly identified responses ranging from 75.3% to 83.7% for lidocaine and from 76.3% to 81.5% for penicillin, with fifth-year students achieving the highest correct response rates.

Regarding risk factors that may worsen a patient’s allergic condition (Q4), stress was recognised by most respondents, with higher proportions among students—83.7% of fifth-year students and 74.2% of third-year students—compared to residents (46.8%) (*p* = 0.001).

Summarising the data presented in Table 3, all participants provided five correct answers (classified as excellent or good). However, third-year students achieved one answer at an excellent level, whereas residents had two answers classified as low.

### 3.3. Participants’ Knowledge About the Management of the Anaphylaxis Treatment

The answers provided by the study participants to the questions in this section are presented in Table 4.

For Question Q5, the correct answer, epinephrine, was recognised by most respondents: 66% of third-year students, 74% of fifth-year students, and 68.1% of residents. More than 80% of participants understood that treatment for anaphylaxis must be initiated as quickly as possible, within 5–10 min (Q6). For Question Q7, regarding the correct dose of epinephrine for adults, residents recorded the highest percentage of correct answers (89.4%), followed by fifth-year students (83.7%) and third-year students (60.8%). The same trend was observed for Q8, where residents again demonstrated the highest proportion of correct answers regarding the route of administration of epinephrine (89.4%), followed by fifth-year students (88%) and third-year students (75.3%). Conversely, the correct site of epinephrine injection (Q9) was known by a lower percentage of respondents: 74.5% of residents, 64.1% of fifth-year students, and 57.7% of third-year students.

Question Q10 concerns the initiation of emergency treatment, which begins with the administration of the drug of choice. This answer was most frequently given by residents (70.2%), followed by fifth-year students (64.1%) and third-year students (52.6%). Knowledge about the medication auto-injector (Q11) was confirmed by 93.6% of residents, 90.2% of fifth-year students, and only 77.3% of third-year students. Epinephrine was identified as the medication contained in the auto-injector by most participants, with percentages ranging from 78.7% (third-year students) to 79.6% (residents) (Q12). The name of the auto-injector, EpiPen (Q13), was correctly identified by a higher proportion of residents (72.3%) compared with students (68.5% among fifth-year students and only 49.5% among third-year students) (*p* = 0.006). Statistically significant differences (*p* = 0.001) were also observed for Question Q14, where the correct answer for secondary medication—namely corticosteroids and antihistamines—was provided by 71.7% of fifth-year students, followed by residents (68.1%) and third-year students (55.7%).

Question Q15 concerns practical experience with anaphylaxis; 26.4% of all participants reported encountering such situations in clinical practice.

Question Q16 was an open-ended query, requiring each participant to specify one or more antihistamine products.

**Figure 1:**
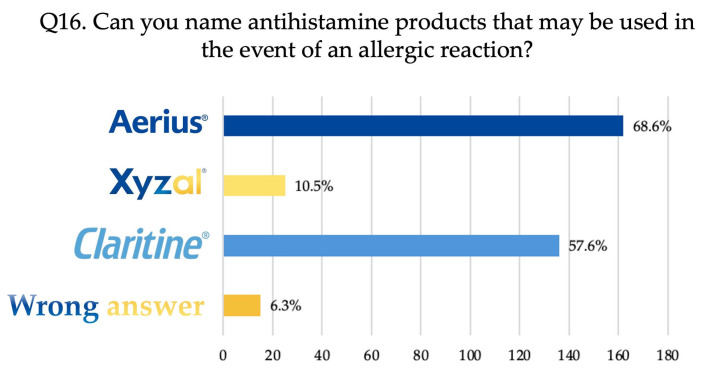
The answers to question Q16: Can you name antihistamine products that may be used in the event of an allergic reaction? (*N* = 236).

As shown in Figure 1, most respondents—162 (68.6%)—named the medication Aerius (desloratadine), 136 (57.6%) mentioned Claritine (loratadine), 25 (10.5%) specified Xyzal (levocetirizine), and 15 (6.3%) gave an incorrect answer.

The last two questions evaluated participants’ confidence in managing an anaphylactic emergency correctly. About 70% of respondents reported feeling unconfident, with a higher proportion among students than residents (76.3% of third-year students, 72.8% of fifth-year students, and 42.6% of residents). This lack of confidence is mainly due to limited practical experience, as shown by 53.3% of third-year students, 57.6% of fifth-year students, and 45% of residents.

The knowledge level for the third section of the questionnaire, shown in Table 5, was classified as follows:

- third-year students: good for Q6, Q8, Q12; moderate for Q5, Q7, Q9, Q10, Q14; and low for Q13;
- fifth-year students: excellent and good for Q5, Q6, Q7, Q8, Q12, Q14; and moderate for Q9, Q10, Q13;
- residents: excellent and good for Q6, Q7, Q8, Q9, Q12, Q13; and moderate for Q5, Q10, Q14.

**Figure 2:**
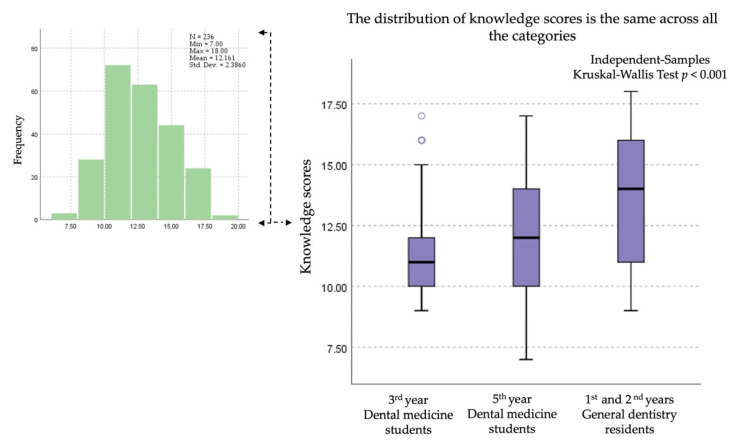
Distribution and variation in anaphylaxis knowledge scores among Dental medicine students and General dentistry residents.

Pairwise comparisons of knowledge scores revealed significant differences across the training levels. After applying the Bonferroni correction, 3rd year students scored significantly lower than general dentistry residents (adj. *p* < 0.001), and 5th year students also scored significantly lower than residents (adj. *p* = 0.008). Although third-year students scored lower than fifth-year students, this difference was not statistically significant (adj. *p* = 0.085) after correction for multiple testing (Figure 2).

## 4. Discussion

Although anaphylaxis is an uncommon occurrence, its severity and potential to be life-threatening necessitate prompt and appropriate management. The responses from the study participants indicate that some students, and even some residents, exhibit a lack of knowledge regarding the symptoms and emergency treatment of anaphylaxis. These deficiencies are more pronounced among students compared to residents, as illustrated in Figure 2.

This situation may be explained by the fact that, in the curriculum of the Faculty of Dental Medicine in Iași, the Medical Emergencies discipline is allocated to the sixth year, despite students beginning clinical activities as early as the third year. During this time, they are introduced to subjects such as Anaesthesiology, Cariology, and Dental Prosthodontics, where they receive their first notions of the risk of anaphylaxis in patients with allergic predispositions. In the fifth year, clinical activities continue with Oral and Maxillofacial Surgery, where students are required to perform minor surgical procedures. In the General Dentistry residency programme, the curriculum does not include courses dedicated to this topic, meaning that all knowledge is acquired during undergraduate studies, supplemented by individual clinical experience. Sixth-year students were not included in the study because medical emergency courses are conducted this year, which would likely have led to the highest rate of correct responses and could have introduced a systematic bias in the comparative analysis.

Although it was observed that the level of knowledge was higher among individuals with greater clinical experience, such as residents, there were notable exceptions. For instance, regarding the question on the first three symptoms of anaphylaxis, facial oedema was regarded by most participants as a pathognomonic sign, with a higher percentage among third-year students (93.8%) compared to residents (85.1%). When compared with other similar studies, facial oedema is widely acknowledged among practitioners. A study conducted in Latin America by Cherrez-Ojeda [29] reported that 85.2% of dentists correctly identified this symptom.

A similar pattern was observed in the question about factors that worsen a patient’s allergic predisposition, where fifth-year students most often recognised stress as a key factor, with statistically significant differences among the three groups. Conversely, the misconception that epinephrine could trigger anaphylactic shock was more common among fifth-year students, indicating gaps in basic pharmacological knowledge.

In Iran, Pakravan [33] observed that dental students had limited knowledge of recognising anaphylactic shock symptoms, with notable differences between younger students, who scored lower, and their older counterparts. Epinephrine is recognised as the primary treatment for this medical emergency, as outlined in international guidelines [8]. No alternative or substitute can replace epinephrine, although systemic corticosteroids and antihistamines may be used to manage severe systemic reactions [34].

In our study, fifth-year students most frequently recognised epinephrine as the preferred medication (74%), followed by residents and third-year students. These findings are consistent with similar research conducted by Vasconcelos [35] in Brazil, Baççioğlu [28] in Turkey, and Algrigri [36] in Saudi Arabia, which reported recognition rates of 78.8%, 70.4%, and 88%, respectively, among dental practitioners and students.

Although anaphylaxis is uncommon, the clinician must respond swiftly to save the patient’s life. The recommended protocol involves: first, stopping exposure to the trigger; assessing breathing and circulation; administering epinephrine; and positioning the patient based on clinical presentation [37]. Epinephrine should be administered via intramuscular injection into the mid-anterolateral thigh (vastus lateralis). The dose for adults and children over 25–30 kg is 0.3 mg, while children under 25 kg require 0.15 mg [30]. Since blood levels peak 10 min after intramuscular injection and decrease by half after 40 min, repeat doses should be given every 5 to 15 min if symptoms persist [22]. An alternative to the standard epinephrine solution is the epinephrine auto-injector (EpiPen), which is convenient, relatively safe, has a lower risk of errors during administration, and allows for quicker delivery. Second-line treatments, such as antihistamines and corticosteroids, manage secondary symptoms but do not replace epinephrine [9].

In Section 2 of the questionnaire, six questions focused on correct anaphylaxis management—dose, route, site of administration, and recognition of the EpiPen. Residents provided the highest number of correct answers, followed by fifth-year and then third-year students. Statistically significant differences were found for the correct epinephrine dose (*p* = 0.001) and for recognising the EpiPen auto-injector (*p* = 0.006). These results suggest that residents greatly benefit from increased clinical experience, maturity, and responsibility towards patients. In our study, 89.4% of residents identified the correct epinephrine dose, a more favourable outcome than in other studies such as Celik [38] and Bulbul [39] in Turkey, where lower rates of 41.7% and 30% were reported.

Nevertheless, the epinephrine injection site remains an area needing improvement for all participants. A large proportion provided incorrect answers, 39.2% of third-year students, 33.7% of fifth-year students, and 23.4% of residents, mistakenly identifying the anterior aspect of the arm. Similar misconceptions have been reported in other countries: in Iraq, Hassan [40] found that 34% of dentists believed epinephrine should be administered subcutaneously or even intravenously (24.5%); in Pakistan, 72.1% of male and 75.7% of female dental students were unaware of the correct route of administration [41]; in Poland, only 27.2% of dentists identified the intramuscular route as the standard for adrenaline injection [42].

The most well-known epinephrine auto-injector is EpiPen^®^, although other brands exist, such as Auvi-Q^®^ in the USA and Allerject^®^ in Canada, often recommended for children [43]. Its popularity is considerable: although commonly included in emergency kits in dental practices, maintaining these kits is costly. In Romania, an epinephrine auto-injector costs approximately €50 and has a shelf life of only 6–8 months. Despite its importance, the auto-injector is not widely available in most countries; only 32% of the world’s 195 nations provide access, mainly high-income countries [8]. In our study, notable differences were observed between groups regarding knowledge of the auto-injector, with more residents than students able to identify EpiPen^®^, highlighting the influence of clinical experience on knowledge retention.

Regarding antihistamine medication, most respondents identified Aerius (desloratadine), followed by Claritine (loratadine) and Xyzal (levocetirizine). In Romania, it is compulsory for every dental practice to have an emergency medical kit containing at least one of these antihistamines, or alternatively Zyrtec (cetirizine). This requirement may explain why practitioners, who are actively involved in clinical work and responsible for maintaining emergency supplies, recognised a greater range of antihistamines than students. However, antihistamines based on cetirizine remain insufficiently recognised.

Concerning confidence in managing anaphylaxis, numerous participants reported feeling unprepared, with notable disparities observed between groups; only 57.4% of residents feel confident in handling such emergencies. Participants attributed this lack of confidence to insufficient practical experience, inadequate specialised training, and limited theoretical knowledge. A study conducted in Latin America identified an even lower percentage (28.7%) of dentists who believed they could manage an anaphylactic episode in their practice [29].

This study showed that participants had limited knowledge of several important aspects. These included how to manage patients with allergic predispositions—many mistakenly believed that allergy testing should take place within dental clinics. Furthermore, misconceptions were noted regarding the initial steps in treating anaphylaxis, with some thinking that airway clearance is the first priority and confusion about the correct site for epinephrine injection. Although over 70% of participants had heard of epinephrine auto-injectors, 43.3% of third-year students and 21.3% of residents could not identify the EpiPen^®^, the device used in emergency kits in Romania.

Although the knowledge level regarding anaphylaxis management was similar between residents and fifth-year students, according to Table 5, there were still differences—residents gave more correct answers to questions about the injection site, the correct dose, and the name of the EpiPen.

This study has several limitations: firstly, the questionnaire focused solely on the clinical presentation and treatment of anaphylaxis and was not comprehensive. Secondly, the study included only students from selected undergraduate years and residents from one residency program (General Dentistry), which does not reflect the knowledge levels among other residency categories. Another limitation is that the findings cannot be generalised to all dental students and residents in Romania, as participants were recruited from a single institution and via convenience sampling.

Although numerous studies exist in the specialised literature that investigate the level of knowledge of dental students/practitioners regarding medical emergencies in the dental office [44,45,46,47,48,49], the present research makes its original contribution by: (i) focusing on anaphylaxis recognition, (ii) including item-level epinephrine management evaluation, (iii) providing comparison across training stages, (iv) providing a baseline data for future multicenter or cross-national benchmarking.

Future research should involve multicentre sampling, students of all undergraduate years, multiple residency specialties, and dentists across experience strata.

The statistical analyses show that the null hypothesis is rejected, indicating significant differences in knowledge levels among the three participant groups.

## 5. Conclusions

The first three symptoms of anaphylaxis—facial oedema, dyspnoea, and severe pruritus—were recognised by many participants; however, students provided more correct answers than residents.

Regarding emergency management, residents demonstrated higher accuracy than students in questions related to the drug of choice, the correct dose of epinephrine, and its appropriate route of administration. Nevertheless, 23.4% of residents still provided incorrect responses concerning epinephrine administration, while a relatively modest proportion (57.4%) expressed confidence in managing an anaphylactic shock in the dental office.

Significant gaps in knowledge were identified regarding the management of anaphylactic shock, such as the incorrect belief that antihistamines are the first-line treatment for anaphylaxis, the misconception that epinephrine should be administered in the arm, and insufficient knowledge regarding the EpiPen auto-injector.

A greater proportion of students than residents reported insufficient experience to manage an acute allergic event in the dental surgery.

Given the critical importance of acute allergic reactions, this study emphasises the need for ongoing educational initiatives and practical training for both students and residents, especially as their academic curricula currently lack structured instruction on managing medical emergencies.

## Figures and Tables

**Table 1:** Characteristics of study participants (*N* = 236).

Variables		Total *N* (%)	Third Year Dental Medicine Students *N* (%)	Fifth Year Dental Medicine Students *N* (%)	General Dentistry Residents *N* (%)
Gender	Male	114 (48.3)	43 (44.3)	39 (42.4)	32 (68.1)
	Female	122 (51.7)	54 (55.7)	53 (57.6)	15 (31.9)
Total *N* (%)		236 (100)	97 (41.1)	92 (39.0)	47 (19.9)
Age (mean ± SD)		24.01 ± 0.39			

SD—standard deviation.

**Table 2:** Participants’ answers to the questions in Section 2-Clinical aspects of anaphylaxis (The correct answers are marked in italics).

Clinical Aspects of Anaphylaxis	Third Year Dental Medicine Students (*N* = 97)	Fifth Year Dental Medicine Students (*N* = 92)	General Dentistry Residents (*N* = 47)	Chi-Square Test
	*N* (%)	*N* (%)	*N* (%)	*p*-Value
Q1. What is your approach when treating a patient with a known history of allergies? (single choice)				
I do not start the treatment during the first appointment	3 (3.1)	6 (6.5)	2 (4.3)	0.033 *
*I refer the patient for a specialist* consultation in the field of allergology	51 (52.6)	44 (47.8)	23 (48.9)	
I perform an allergy test in the dental office	43 (44.3)	42 (45.7)	19 (40.4)	
I treat the patient immediately	0	0	3 (6.4)	
Q2. What are the first three symptoms that may appear in an anaphylactic shock?				
Vomiting	22 (22.7)	35 (38.0)	13 (27.7)	0.065
*Facial oedema*	91 (93.8)	80 (87.0)	40 (85.1)	0.175
Headache	36 (37.1)	31 (33.7)	27 (57.4)	0.020 *
*Dyspnoea*	77 (79.4)	71 (77.2)	38 (80.9)	0.868
*Severe pruritus*	63 (64.9)	64 (69.6)	36 (74.5)	0.498
Arterial hypotension	59 (60.8)	50 (54.3)	29 (61.7)	0.586
Nausea	38(39.2)	44 (47.8)	19 (40.4)	0.454
Vertigo	38 (39.2)	49 (53.3)	18 (38.3)	0.095
Q3. Which of the following substances can trigger an anaphylactic shock? (multiple choice)				
Epinephrine	13 (13.4)	24 (26.1)	11 (23.4)	0.081
*Xylocaine*	73 (75.3)	77 (83.7)	36 (76.6)	0.335
Antihistamines	8 (8.2)	6 (6.5)	1 (2.1)	0.368
*Penicillin*	74 (76.3)	75 (81.5)	36 (76.6)	0.646
Glucose	1 (2.1)	0	1	0.236
Q4. Which factors can aggravate the allergic predisposition of a patient? (multiple choice)				
Alcohol consumption	47 (48.5)	36 (39.1)	19 (40.4)	0.395
*Stress*	72 (74.2)	77 (83.7)	22 (46.8)	0.001 *
*Infections*	61 (62.9)	51 (55.4)	30 (63.8)	0.491
Smoking	48 (49.5)	36 (39.1)	22 (46.8)	0.345
I don’t know	3 (3.1)	0	0	0.113

* Significance level of 0.05 (Chi-square test).

**Table 3:** Knowledge level of the study participants for the questions in the first section of the questionnaire.

Items		Third Year Students	Fifth Year Students	General Dentistry Residents
Q1		moderate	low	low
Q2	facial oedema	excellent	good	good
	dyspnoea	good	good	good
	severe pruritus	moderate	good	good
Q3	xylocaine	good	good	good
	penicillin	good	good	good
Q4	stress	good	good	low
	infections	moderate	moderate	moderate

**Table 4:** Responses to the questions regarding the management of anaphylaxis (The correct answers are marked in italics).

Management of Anaphylaxis	Third Year Dental Medicine Students (*N* = 97)	Fifth Year Dental Medicine Students (*N* = 92)	General Dentistry Residents (*N* = 47)	Chi-Square Test
	*N* (%)	*N* (%)	*N* (%)	*p*-Value
Q5. Which is the drug of choice in the treatment of anaphylactic shock?				
Corticosteroids	13 (13.4)	12 (13.0)	4 (8.5)	0.417
Antihistamines	18 (18.6)	12 (13.0)	11 (23.4)	
*Epinephrine*	64 (66.0)	68 (74.0)	32 (68.1)	
I don’t know	2 (2.0)	0	0	
Q6. After how long from the onset of symptoms should treatment be initiated?				
*0–5 min*	80 (82.4)	89 (96.7)	45 (95.7)	0.009 *
5–15 min	5 (5.2)	0	0	
15–30 min	5 (5.2)	0	0	
I do not know	7 (7.2)	3 (3.3)	2 (4.3)	
Q7. What is the correct dosage of Epinephrine to be injected in an adult?				
*0.3 mg*	59 (60.8)	77 (83.7)	42 (89.4)	0.001 *
1 mg	26 (26.8)	7 (7.6)	5 (10.6)	
2 mg	4 (4.2)	1 (1.1)	0	
I don’t know	8 (8.2)	7 (7.6)	0	
Q8. What is the route of administration for Epinephrine?				
*Intramuscular*	73 (75.3)	81 (88.0)	42 (89.4)	0.214
Subcutaneous	13 (13.4)	7 (7.6)	4 (8.5)	
Oral	9 (9.3)	3 (3.3)	1 (2.1)	
I don’t know	2 (2.0)	1 (1.1)	0	
Q9. Where should Epinephrine be injected?				
Intramuscularly, in the anterior aspect of the arm	38 (39.2)	31 (33.7)	11 (23.4)	0.416
*Intramuscularly, in the outer part of the thigh*	56 (57.7)	59 (64.1)	35 (74.5)	
I don’t know	3 (3.1)	2 (2.2)	1 (2.1)	
Q10. What is the first intervention in the case of an anaphylactic shock?				
*Injection of the drug of choice*	51 (52.6)	59 (64.1)	33 (70.2)	0.074
Airway clearance	29 (29.9)	18 (19.6)	8 (17.0)	
Placing the patient in the Trendelenburg position	6 (6.2)	1 (1.1)	0	
Calling for an ambulance	11 (11.3)	14 (15.2)	6 (12.8)	
Q11. Have you ever been informed about auto-injectable medications?				
Yes	75 (77.3)	83 (90.2)	44 (93.6)	0.009 *
No	22 (22.7)	9 (9.8)	3 (6.4)	
Q12. If you answered “Yes” to the previous question, please specify which ones.				
*Epinephrine*	59 (78.7)	69 (83.1)	35 (79.6)	0.962
Cortisone	13 (17.3)	11 (13.3)	7 (15.9)	
I don’t know	3 (4.0)	3 (3.6)	2 (4.5)	
Q13. Can you name an auto-injectable product?				
EpiPen^®^	48 (49.5)	63 (68.5)	34 (72.3)	0.006 *
I don’t know	42 (43.3)	19 (20.7)	10 (21.3)	
Wrong answer	7 (7.2)	10 (10.8)	3 (6.4)	
Q14. Which are the secondary medications administered in anaphylactic shock?				
Theophylline + Cortisone	13 (13.4)	1 (1.1)	0	0.001 *
*Cortisone + Antihistamines*	54 (55.7)	66 (71.7)	32 (68.1)	
Cortisone + Antihistamines + Theophylline	30 (30.9)	25 (27.2)	15 (31.9)	
Q15. Have you ever encountered patients who developed an allergic reaction during treatment?				
Yes	8 (8.2)	7 (7.6)	5 (10.6)	0.827
No	89 (91.8)	85 (92.4)	42 (89.4)	
Q16. Can you name antihistamine products that may be used in the event of an allergic reaction?				
Aerius	63 (64.9)	63 (68.5)	36 (76.6)	0.368
Xyzal	15 (15.5)	3 (3.3)	7 (14.9)	0.014 *
Claritine	45 (46.4)	57 (62)	34 (72.3)	0.007 *
Wrong answer	10 (10.3)	3 (3.3)	2 (4.3)	0.112
Q17. Do you feel confident in managing an anaphylactic shock in the dental office?				
Yes	23 (23.7)	25 (27.2)	27 (57.4)	<0.001 *
No	74 (76.3)	67 (72.8)	20 (42.6)	
Q18. If you answered “No” to the previous question, what is the reason?				
I do not have sufficient knowledge	7 (9.3)	5 (7.5)	1 (5.0)	0.890
I have not attended any training sessions on medical emergencies	5 (6.7)	6 (9.1)	2 (10.0)	
I lack practical experience	40 (53.3)	38 (57.6)	9 (45.0)	
All above	23 (30.7)	17 (25.8)	8 (40.0)	

* Significance level of 0.05 (Chi-square test).

**Table 5:** Knowledge level of study participants regarding the management of anaphylaxis.

Items	Third Year Students	Fifth Year Students	General Dentistry Residents
Q5	moderate	good	moderate
Q6	good	excellent	excellent
Q7	moderate	good	good
Q8	good	good	good
Q9	moderate	moderate	good
Q10	moderate	moderate	moderate
Q12	good	good	good
Q13	low	moderate	good
Q14	moderate	good	moderate

## Data Availability

The original contributions presented in this study are included in the article. Further inquiries can be directed to the corresponding author.
